# General Anesthetic-Induced Neurotoxicity in the Immature Brain: Reevaluating the Confounding Factors in the Preclinical Studies

**DOI:** 10.1155/2020/7380172

**Published:** 2020-01-07

**Authors:** Ailin Luo, Xiaole Tang, Yilin Zhao, Zhiqiang Zhou, Jing Yan, Shiyong Li

**Affiliations:** Department of Anesthesiology, Tongji Hospital, Tongji Medical College, Huazhong University of Science and Technology, 1095 Jiefang Avenue, Wuhan 430030, Hubei, China

## Abstract

General anesthetic (GA) is used clinically to millions of young children each year to facilitate surgical procedures, relieve perioperative stress, and provide analgesia and amnesia. During recent years, there is a growing concern regarding a causal association between early life GA exposure and subsequently long-term neurocognitive abnormalities. To address the increasing concern, mounting preclinical studies and clinical trials have been undergoing. Until now, nearly all of the preclinical findings show that neonatal exposure to GA causally leads to acute neural cell injury and delayed cognitive impairment. Unexpectedly, several influential clinical findings suggest that early life GA exposure, especially brief and single exposure, does not cause adverse neurodevelopmental outcome, which is not fully in line with the experimental findings and data from several previous cohort trials. As the clinical data have been critically discussed in previous reviews, in the present review, we try to analyze the potential factors of the experimental studies that may overestimate the adverse effect of GA on the developing brain. Meanwhile, we briefly summarized the advance in experimental research. Generally, our purpose is to provide some useful suggestions for forthcoming preclinical studies and strengthen the powerfulness of preclinical data.

## 1. Introduction

General anesthetic (GA) has been used for more than a century in clinical anesthesia. Each year GA are administered to millions of young children to facilitate the invasive examination or surgical procedures and relieve the perioperative discomfort. However, concerns over the neurotoxic effect of GA on the developing brain that contributes to long-term neurodevelopmental sequelae have been burgeoning during the past two decades. The concern mainly arose from the experimental findings that suggested *γ*-aminobutyric acid A (GABAA) receptor agonists and/or *N*-methyl-D-aspartic acid (NMDA) receptors antagonists could induce significant neuroapoptosis and long-term deficit in learning and memory [1, 2]. As known, GABAA and NMDA receptors are widely accepted targets of commonly used GA [3, 4], although the precise mechanism of GA still needs to be further elucidated. Heretofore concern about GA-related neurotoxicity in the developing brain has become an important issue for parents, clinical practitioners, and healthcare providers.

Since the profound findings showed that ketamine and alcohol elicited extensive neuronal apoptosis in the brain of postnasal day (PND) 7 rats and prolonged spatial memory and learning disorder [1, 2], studies to investigate the potentially neurotoxic effect of the developing brain increased in an outburst trend. Among this research, a landmark study in this field showed that PND7 rats exhibited neuroapoptosis in a variety of brain regions and prolonged impairment in learning and memory after exposed to a commonly used anesthetic cocktail (N_2_O-midazolam-isoflurane) for 6 h [5]. Subsequent preclinical studies demonstrated that GA caused a vast amount of acute injury and long-term cognitive impairments in rodents [6–13] and nonhuman primates (NHP) [9, 10, 14–23]. Even worse, exposure to GA in neonatal rodents produced adverse effects on the learning and memory of their offspring via epigenetic modulation [24–27]. Taken together, these solid preclinical findings suggest that exposure of the developing brain to GA contributes to the long-term adverse neurodevelopmental outcome.

In this context, a recent U.S. Food and Drug Administration (FDA) issued the warning that prolonged or repeated exposure to GA in children younger than 3 years or in pregnant women during their third trimester may affect neurodevelopment in children and advised a change in labeling regarding the safe use of anesthetic and sedative agents [28]. Frankly speaking, this warning fueled the controversy whether neonatal GA exposure could cause long-term neurodevelopmental impairment, as until now, there is no definite clinical evidence establishing a causal link between early life GA exposure to prolonged neurobehavioral abnormalities. The FDA's warning is questioned by some scholars and the American College of Obstetricians and Gynecologists (ACOG) [29–31]. Moreover, the ACOG presents their special concern related to the use of GA in pregnant women and pointed out the limitations of data that support the FDA's announcement. Furthermore, the findings of several influential clinical trials are sequentially published and none of them suggest a causal association between early life GA exposure and adverse long-term neurodevelopmental outcomes [32–37]. The significant discrepancy between the preclinical data and the clinical findings does not only relate to the inherent uncertainty to translate the experimental findings into humans but also relate to the confounding factors that weaken the powerfulness of the data from the preclinical research. Meanwhile, the confounding effect of early social stress in the clinical trials, such as maternal separation and social isolation or defeat [38–41], on the neurodevelopment possibly causes the discrepancy between preclinical and clinical studies. As it is beyond the scope of the present work, we do not discuss all of them in the present review. Therefore, in the present review, we delved into the previous preclinical studies and tried to find out the potential confounding factors.

## 2. Progress in Experimental Research

### 2.1. Advance in Rodents

The side effects of GA on the brain came into sight of researchers in the 1970s. It was first observed that diethyl ether and halothane induced brain injury if they diffused directly into the brain [42]. Next, several studies showed that rats during early development, but not adult rats, exposed to the environmental concentration of halothane in operating rooms caused synaptic abnormalities and detrimental effects in learning abilities [43, 44]. Later, it was established that the sensitive exposure timing of halothane to rats was the second trimester, when the organogenesis was occurring, and that halothane exposure during this period induced persistent learning deficit in the adulthood of the offspring [45]. Till then, the topic about the potentially detrimental effect of GA on the developing brain was not a widespread issue. What pushed this topic under the spotlight was the finding that alcohol or ketamine triggered extensive neuroapoptosis in rats at PND7 as well as long-term spatial learning and memory disorders [1, 2]. Meanwhile, it was established that the peak vulnerability of developing brain to NMDA antagonists or GABA agonists was the brain growth spurt period, which was also deemed as the enhanced vulnerability to nutritional and other growth restrictions [46]. The timing of the brain growth spurt occurs in different mammalian species at different times in relation to birth. In rats, it is from 1 to 2 days before birth to 1 to 2 weeks after birth; in the nonhuman primate (NHP), it is from about the second trimester to 5 weeks after birth, while in humans it is from the 6th month of gestation to the early stage of infancy [46, 47]. In the following experimental studies, this timing of the brain growth spurt is considered as the vulnerable time window in which the developing brain was sensitive highly to GA.

The work by Jevtovic-Todorovic, in which PND7 rats were exposed to an anesthetic combination of isoflurane, nitrous oxide, and midazolam for 6 hours, showed extensive neuronal apoptosis in the brain and persistent impairments in spatial memory, which opened the door to this new field [5]. Subsequently, there was a boom in the studies showing that GA, such as sevoflurane, desflurane, propofol, etomidate, and midazolam, induced developmental neuroapoptosis via various pathophysiological mechanisms. Furthermore, GA were also found to induce various types of neurodegeneration, such as apoptosis of oligodendrocytes and neurons, microglial activation, cytoskeletal abnormalities of astrocytes, imbalanced differentiation of neural precursor cells, disruption of mitochondrial dynamics, and even damage to the blood-brain barrier. Because the neurodegenerative changes and potential injury mechanisms were thoroughly and elaborately outlined in recently published reviews [6–8, 11], we will not repeat it in the present paper.

### 2.2. Advances in NHPs

The findings from rodent models could not be directly extrapolated to clinical practice because of the species differences. In support of the hypothesis that GA might induce neurotoxicity in the developing brain of humans, increasing studies were carried out in NHPs. Slikker et al. found that 3 hours of ketamine anesthesia induced neuronal apoptosis in 5-day-old rhesus monkeys [20]. Brambrink et al. found that 1.5% isoflurane treatment for 5 hours induced neuronal apoptosis in 6-day-old rhesus monkeys [14]. Paule et al. found that ketamine anesthesia for 24 hours led to long-term cognitive impairments in 7-day-old rhesus monkeys [19]. As shown in rodents, neonatal exposure to sevoflurane, isoflurane, nitric oxide, propofol, ketamine, and others was found to induce apoptosis of neurons and oligodendrocytes and long-term cognitive impairment in rhesus monkeys [10, 14–22, 48]. Notably, a new important advance in this field was that recent findings showed that GA disrupted myelin formation in the developing brain of NHPs and mice [49, 50]. Zhang et al.'s report was the first finding that sevoflurane exposure in early life disrupted folate metabolism and then interrupted oligodendrocyte development in NHPs [50]. Indeed, these experimental data obtained from NHPs confirmed those from rodents. Importantly, because NHPs are highly similar to humans in terms of developmental timing and duration and complexity of brain development, these data indicated that GA might affect the development of infants or even long-term cognitive function.

## 3. Potential Challenges in the Experimental Setting

### 3.1. Uncertainty of the Incidence of Cognitive Damage

The incidence of long-term cognitive impairments was not identified in rodent or NHP models. As known, not every animal in the GA treatment group showed neuronal damage or long-term cognitive impairments. Hence, only after determining the probability range of the cognitive decline of GA can we further set the appropriate sample size when conducting the study.

### 3.2. Effect of Surgical Stress on the Developing Brain

Anesthesia and surgery or invasive procedures are necessarily interconnected in clinical practice. Anesthesia is primarily designated to facilitate the surgical procedures and reduce the stress of surgical stress, so an integrated evaluation of the effects of general anesthesia and surgery on the developing brain represents the real-world clinical situation. A prospective study showed that the effect of midazolam was weaker than that of surgery on the hippocampal development in preterm infants who underwent surgery [51]. Noncardiac surgery in preterm infants (<42 weeks) was an independent risk factor for cognitive impairments at the age of 3 to 6 years [52]. These findings suggest that surgery weighs over anesthesia to affect the cognitive performance in infants. In line with the clinical findings, the preclinical models show that intraplantar injection of complete Freund's adjuvant was found to reduce ketamine-induced neuronal apoptosis in the PND7 brain [53]. In turn, ketamine reduces cell death following inflammatory pain in the newborn rat brain [54, 55]. However, neonatal rodents exposed to only ketamine without noxious stimuli displayed acute neural injury and long-term cognitive impairment [56–58]. These data suggest that whether ketamine or other GA are toxic or protective to the developing brain depends on the context with or without noxious stimuli. Taken together, these observations suggest that the evaluation of the effects of GA on the developing animal brain without surgery probably overestimates the developmental neurotoxicity of GA.

### 3.3. Varied Regimens of GA Exposure

The varied regimens of exposure to GA are particularly obvious in rodent studies. The pattern adopted in experimental research varies in the different research groups. Jevtovic-Todorovic et al. first used the protocol that PND7 rat pups were exposed to three commonly used GA as a cocktail for 6 hours [6]. Subsequently, the duration of anesthesia changed into 4 hours or 2 hours. In addition, the concentrations of isoflurane varied from 2%, to 1.8%, 1.5%, or 1.4% in different study groups. In fact, the minimum alveolar concentration (MAC) values of rats changed in a specific pattern during postnatal maturation [59]. In a word, if unanimous regimens of GA exposure were detected at specific postnatal days, the credibility of findings derived from experimental studies would be improved.

### 3.4. The Physiological Disturbance of Anesthesia

In experimental models, nearly all studies have included arterial blood gas analysis to exclude the possibility that an anesthesia-induced physiological disturbance and other factors, such as high oxygen concentrations and abnormal temperature, led to neuronal injury [5, 60–62]. However, this viewpoint was challenged in a NHP model [63]. Furthermore, a newly published study found that PND7 mice exposed to 1.5% isoflurane (or 3.5% sevoflurane) for 2 hours showed respiratory depression, hypercarbia, and acidosis [64], of which the finding was similar to a previous study [65]. Furthermore, the data showed that neuroapoptosis synchronized with the onset of severe systemic metabolic derangements. On the other hand, previous studies showed that carbon dioxide treatment, which mimicked respiratory depressant effects of anesthesia, induced neuroapoptosis but improved the working memory [66]. Surprisingly, a recent study also suggested that isoflurane-induced developing neuroapoptosis was not indispensable for its induction of prolonged cognitive impairment with neonatal exposure to isoflurane [48]. These findings suggest the neuroapoptosis resulted from the physiological perturbations may not be the principal cause of long-term cognitive impairment. However, we need to cautiously evaluate the confounding effect of anesthesia-induced physiological perturbations on the neurobehavioral performance of neonatal subjects exposed to GA.

### 3.5. Further Updating of the Potentially Injurious Mechanisms

Caspase-3 activation is identified as the main marker of GA-induced neuroapoptosis and a principal mechanism which mediates the long-term neurodevelopmental impairment of GA in a larger number of preclinical studies. However, recent studies found that caspase-3 activation is involved in neuronal physiological long-term depression (LTD) and pathological synaptic dysfunction without causing apoptosis [67, 68]. This suggests that caspase-3 may play a role in the physiological process or the nonapoptotic pathological injury. Furthermore, a recent study showed that inhibiting isoflurane-induced neuronal apoptosis cannot improve spatial memory [48]. It was implicated that neuroapoptosis did not contribute to long-term cognitive dysfunction induced by 2% sevoflurane in rats at postnatal day 7 [69]. As there is physiological neuroapoptosis with the maturing process of neurons in the developing brain [70], it needs further investigation whether GA-induced caspase-3 activation is apoptotic or nonapoptotic (including physiological and pathological) in developing neuron and whether a causal relationship exists between caspase-3 activation and long-term cognitive impairment in the future study. Meanwhile, as the endogenous apoptotic pathways are synaptic activity dependent [71, 72], it is necessary to ascertain whether GA-induced caspase-3 activation is associated with the inhibitory effect of GA on some type of synapse and is reversible after its elimination.

### 3.6. Side Effect of Adjuvants

Under experimental conditions, adjuvants are almost inevitable, without exception to the field we discuss. Dimethyl sulfoxide (DMSO), a commonly used solvent in experimental studies, caused extensive neuroapoptosis at the developmental stage [73]. Although the concentrations of DMSO used in research on the developmental neurotoxicity induced by GA are lower than 0.5% [5, 74–76], it remains unclear whether a low concentration of DMSO causes a deterioration of GA-induced developmental neurotoxicity. In addition, Ca^2+^ indicators (Fluo-4AM, Fluo-2AM, Rhod-2AM) that were used to investigate the mechanism of GA-induced neurotoxicity [77–81] directly inhibited the activity of the Na^+^-K^+^ adenosine triphosphate enzyme and spontaneous calcium signaling in neurons and astrocytes [82]. Therefore, more attention might be paid to weighing the effect of auxiliary reagents or indicators on developmental neurons. Actually, the elaborate adjuvants' control in the experiment will eliminate, or at least reduce, the confounding effect of adjuvants.

### 3.7. Gender-Dependent Effect

The regional difference in the precocious developmental process of the brain indeed exists between males and females. This means that we should weigh the gender-dependent effect when we determine the potential impact of neurotoxic agents on the neurodevelopmental outcome. However, gender factor is not taken into consideration in the field of GA neurotoxicity on neurodevelopment until the finding showed that neonatal isoflurane exposure at postnatal day 7 induced long-term cognitive dysfunction in male but not female rats [83]. A recent study reported that the vulnerability window of isoflurane exposure for female rats was at postnatal day 4, while that for male rats was at postnatal day 7 [84]. It was suggested that the differential timing in peak susceptibility of the developing brain to isoflurane was correlated with sex-specific expression of chloride cotransporters NKCC1 and KCC2, known to regulate the excitatory-to-inhibitory conversion of GABA_A_ receptor in the early postnatal period and mediate the sex-dependent functional shift of GABA_A_ receptor in developing hippocampus [85]. This challenged the widely adopted standpoint that the vulnerable timing of GA exposure was at postnatal day 7, which was established without differentiation between sexes [5]. In addition, KCC2 was found to be associated with propofol-induced alternation of the synaptic structure without considering the factor of sex [86]. Collectively, overlooking the gender-dependent difference in the vulnerability window of GA would result in misunderstanding the effect of GA on neurodevelopmental outcomes.

### 3.8. Data Reproducibility

Data reproducibility has always been a focus of scientific research. In the 2017 special issue entitled “Anaesthetic Neurotoxicity and Neuroplasticity” of the *British Journal of Anaesthesia*, two articles examining the effect of dexmedetomidine on sevoflurane-induced developmental neurotoxicity were published. One study found that 1 *μ*g/kg dexmedetomidine did not alleviate sevoflurane-induced neuronal apoptosis during development, and higher concentrations of dexmedetomidine induced neuroapoptosis in hippocampal CA3 and the ventral thalamic nucleus [87]. However, another study found that 1 *μ*g/kg dexmedetomidine reduced sevoflurane-induced neuroapoptosis [88]. These studies showed paradoxical results but adopted the same experimental protocol. The journal also published an editorial on data repeatability [89]. In 2009, Sanders et al. first reported that 25 *μ*g/kg dexmedetomidine reduced developmental neuroapoptosis induced by 0.75% isoflurane [90], but Liu et al. found that a cumulative dose >50 *μ*g/kg (five doses in 6 hours) caused developmental neuroapoptosis [91]. Assuming that drug treatment protocol was absolutely standardized, the concern has to switch to the sensitive topic we mentioned at the beginning of this paragraph.

## 4. Conclusions

The issue regarding a relevant association between early life GA exposure and the adverse long-term neurodevelopmental outcome has been under debate for nearly two decades. Although great efforts have been made worldwide to draw a reasonable conclusion, the neuropathological mechanism underlying GA-induced neurotoxicity has not yet been fully elucidated. In light of that, a series of well-designed clinical studies have shown that GA exposure during early life is not causally associated with the long-term neurodevelopmental outcome. Preclinical studies would be critically and prudently conducted to exclude the effect of confounding factors on the principal findings. On the other hand, we could not totally neglect the undesirable effect of GA on the developing brain. As the Chinese proverb says “every medicine has its side effect.” Newly published data, extracted from the MASK study and analyzed by factor and cluster analyses, showed that multiple exposures to general anesthesia before the age of 3 years were associated with specific deficits in neuropsychological tests [92]. Therefore, the forthcoming well-designed clinical studies will provide some useful information, such as which factors might predict the risk and which strategies might reduce the risk.
